# Negative and Cognitive Symptoms of Schizophrenia: Are there Adequate Pharmacological Treatments?

**DOI:** 10.1192/j.eurpsy.2022.2005

**Published:** 2022-09-01

**Authors:** O. Vasiliu

**Affiliations:** Dr. Carol Davila University Emergency Central Military Hospital, Psychiatry, Bucharest, Romania

**Keywords:** cognitive symptoms, schizophrénia, negative symptoms

## Abstract

**Introduction:**

The ongoing debate regarding the treatment efficacy for negative and cognitive symptoms in schizophrenia is a subject of continuous frustration among psychiatrists. These symptoms are reputedly hard to tackle therapeutically and their impact on long-term functional prognosis made them an important target for the maintenance treatment.

**Objectives:**

To review the literature in order to find the most adequate treatment options for negative and cognitive symptoms of schizophrenia.

**Methods:**

A literature review was performed through the main electronic databases (PubMed, CINAHL, SCOPUS, EMBASE, www.clinicaltrials.gov) using the search paradigm “schizophrenia” AND “negative symptoms” AND “cognitive symptoms” AND “pharmacological treatment”. All papers published between January 2000 and August 2021 were included.

**Results:**

The efficacy of atypical antipsychotics over cognitive dysfunctions in schizophrenia decreases with time, without significant differences between the agents. Clozapine, amisulpride, olanzapine, and risperidone have superior efficacy over positive and negative symptoms, with small to moderate effect sizes. Meta-analyses show decreased severity of negative symptoms during treatment with atypical antipsychotics, especially with clozapine, amisulpride, olanzapine, zotepine, and risperidone. Atypical antipsychotics had a superior effect over neurocognitive domains when compared to the typical antipsychotics. Newer atypical antipsychotics, with partial D2/D3 agonism, are preferred to other atypical agents, although based on a low level of evidence. Antidepressants, especially mirtazapine, may be a solution for negative symptoms, while modafinil/armodafinil is also useful as an add-on.

**Conclusions:**

Although new therapeutic options are explored, there is a paucity of encouraging results from the pipeline regarding the treatment efficacy over negative/cognitive symptoms.

**Disclosure:**

No significant relationships.

